# Anterior Fontanelle Wormian Bone/ Fontanellar Bone: A Review of this Rare Anomaly with Case Illustration

**DOI:** 10.7759/cureus.1443

**Published:** 2017-07-07

**Authors:** Jaspreet Johal, Joe Iwanaga, Marios Loukas, R. Shane Tubbs

**Affiliations:** 1 Department of Anatomical Sciences, St. George's University School of Medicine, Grenada, West Indies; 2 Seattle Science Foundation; 3 Neurosurgery, Seattle Science Foundation

**Keywords:** cranial fontanelles, cranial sutures, wormian bone, craniosynostosis, bony dysplasia, skeletal anomaly, accessory bone

## Abstract

Wormian bones are a relatively rare skeletal anomaly that present as accessory bone(s) within the sutures of the cranium and even more rarely within the fontanelles. It is believed that they arise from the formation of abnormal cranial ossification centers. Although not extensively reported in the literature, this anomaly is thought to be associated with other anatomical anomalies such as osteogenesis imperfecta, rickets, and other bone dysplasias. When located within the fontanelles, the most likely site of occurrence is the posterior fontanelle. This case report describes a rare incidence of wormian bone within the anterior fontanelle of an infant who had concurrent craniosynostosis. More research and reporting of this anomaly is necessary to verify any correlated syndromes, and determine the underlying etiology.

## Introduction

Wormian bones occur as small, accessory bones within the cranial sutures and fontanelles. The skull itself is composed of several flat bones that fuse together after birth. These sites of fusion are the bony sutures in which wormians most commonly occur [1-2]. Historically, the term “wormian” was coined by Thomas Bartholin in honor of the Dutch anatomist Olaus Wormius [1]. Etiologically, they are believed to arise from abnormal ossification centers within the cranium [1,3], which are thought to form in addition to those that are normally present during cranial development, giving rise to supernumerary bones within the skull [1]. Wormian bones can be considered an abnormal anatomical variant, although they occur more frequently in certain bone dysplasia conditions including osteogenesis imperfecta, rickets, cleidocranial dysostosis, and pycnodysostosis [3-4]. A recent analysis showed that around half of all patients in a pediatric population aged 0-3 years had at least one wormian bone, mostly in the lambdoid suture [4]. Another review concluded that around 8%-15% of the population have at least one wormian bone [2]. Other common locations include the coronal suture, along with the bregma, lambda, and pterion [1-2]. Wormian bones are more likely to occur unilaterally on the right side of the cranium [1].

Clinically, some pathological and diagnostic value has been attributed to wormian bones. One recent study suggested that their presence within the pediatric population could clarify the origins of unexplained bone fractures, helping to distinguish between conditions associated with wormian bones such as osteogenesis imperfecta and other causes such as physical abuse [4]. Their presence could also help in diagnosing more hidden disorders that would otherwise go unnoticed [1]. In the more pathologically significant cases, there are least ten wormian bones larger than around 6 mm x 4 mm and arranged in a mosaic-type pattern [1]. A number of environmental and genetic factors have been associated with wormian bones, although the factors most predictive of this anomaly are still uncertain. They are more likely to occur in individuals with crania that are circumferentially anteroposteriorly deformed and have also been associated with a number of genetic syndromes [2]. They are also more common in the Chinese population than other demographic groups [2].

Wormian bones are relatively rare within fontanelles, the incidence being greater in the posterior than the anterior fontanelle [1]. The anterior fontanelle is an integral part of the pediatric exam, and any abnormalities can indicate an underlying condition [5]. The fontanelles are at the junctions of the bony sutures, the anterior one lying at the convergence of the sagittal, metopic, and coronal sutures [6]. It is normally expected to close by around 12 to 18 months via ossification of the surrounding bones [6]. Failure of this closure can reflect a broad range of underlying pathologies [6]. A review of the available literature shows few reports of the occurrence of wormian bones within the anterior fontanelle, highlighting the significance of the case described below.

## Case presentation

**Figure 1:**
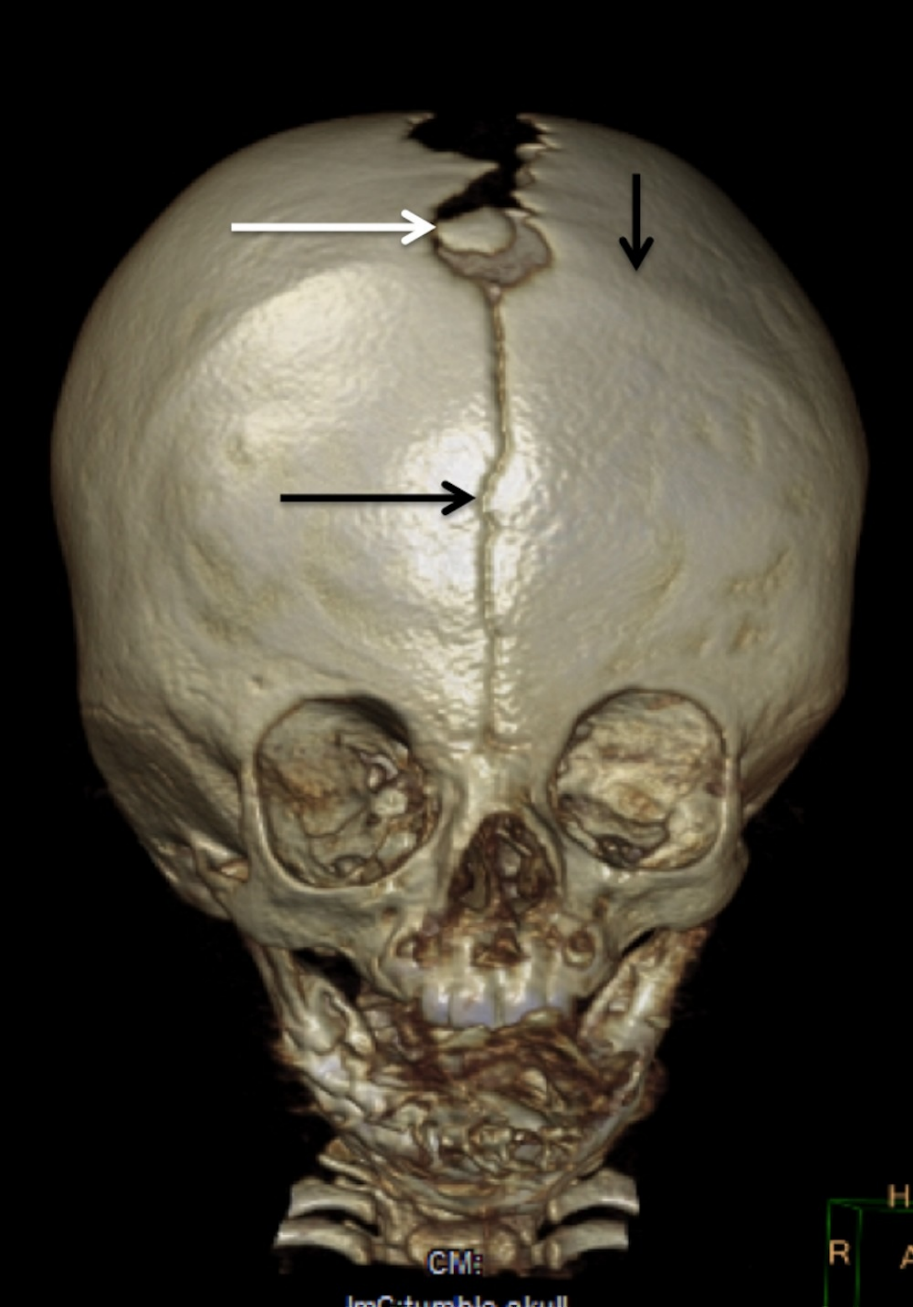
Anterior fontanellar wormian bone in a six-month-old male infant 3D computed tomography (CT) noting bilateral coronal synostosis (vertical arrow over synostosed left black coronal suture), metopic suture (lower horizontal black arrow), and anterior fontanelle wormian bone (middle white arrow).

A six-month-old male presented with small head circumference (<25th percentile) and hypotelorism. No other health related issues were identified in this child with a normal gestation and the second baby to this family who was non-consanguineous. On exam, the patient was noted to have the previously identified mild hypotelorism. In addition, ridges were palpated along the left and right coronal sutures. A metopic suture was also palpated on examination. The sagittal suture had not closed. Anteriorly, the anterior fontanelle was open and not tense. However, a bony island was palpated in the center of the anterior fontanelle. Imaging confirmed the bilateral coronal synostosis, metopic suture, open sagittal suture, and wormian bone of the anterior fontanelle (Figure 1).

The child’s further physical examination was within normal limits. Follow-up visits and examinations did not reveal any issues with development or fulfillment of appropriate milestones. The infant displayed no signs of cognitive delay or abnormalities of physical development. The child did not meet the requirement for any diagnosable or recognized developmental syndrome. At seven months old, the patient underwent operative release of the left and right coronal sutures. At operation, the floating bone (wormian bone) within the anterior fontanelle was noted and removed without consequences although it was firmly attached to the underlying dura mater and superior sagittal sinus. At one-year followup, the patient is doing well with no new complaints from the parents and is developmentally normal. 

## Discussion

As mentioned above, wormian bone deformities within the anterior fontanelle are relatively rare since wormian bones are more common within the cranial sutures. A recent review found that abnormal ossifications within the anterior fontanelle are more likely in association with concurrent craniosynostosis, sagittal synostosis being the most commonly implicated site [6]. It has been suggested that wormian bones within the posterior segments of the skull are more related to environmental factors than those within the anterior segments [7]. Possible environmental factors include traditional supine positioning of infants during sleep in Chinese familes, which may contribute to increased incidence of brachycephalic deformations in Chinese infants compared to infants from other parts of the world [7]. Multiple analyses have suggested that they can be used as diagnostic indicators of more severe syndromes of the central nervous system or bone development [7-8].

The relative rarity of wormian bones within the anterior fontanelle makes it difficult to establish any specific association between this occurrence and other syndromes. A recently published case report suggested a novel association between wormian bones involving the anterior fontanelle and severe facial dysmorphisms and midline abdominal defects [9]. Reid and colleagues described a neonate of Afghani origin who was born with a wormian bone in the anterior fontanelle, along with facial dysmorphic anomalies resulting in exophthalmos [9]. As this is a previously unreported association, it could either be a novel correlation or a concurrence of unrelated anomalies within the same patient. Given the rarity of reported cases of anterior fontanellar bones, more data are needed before any conclusion about associations between this anomaly and other developmental syndromes can be drawn.

## Conclusions

Wormian bones of the anterior fontanelle are rare. As seen in our case, they can be associated with craniosynostosis. At operation, surgeons should be aware that such an anomalous bone can be firmly attached to the underlying superior sagittal suture via its dural connections. A review of the available literature shows that there is a scarcity of presently available case reports of this anomaly, and varying reports of their frequency and etiology. More reporting of the anomaly would clarify the uncertainty surrounding this anomaly. Even more rare are reports of wormian bones being found within fontanelles as opposed to within suture lines. It is possible that a varying etiology exists for these different locations, and they may also have distinct associations with other clinical syndromes and anatomical anomalies. It is recommended that a more comprehensive review of the subject contrast individual case reports to better decipher the etiology of this deformity.
